# Adult age differences in prospective memory in the laboratory: are they related to higher stress levels in the elderly?

**DOI:** 10.3389/fnhum.2014.01021

**Published:** 2014-12-23

**Authors:** Andreas Ihle, Matthias Kliegel, Alexandra Hering, Nicola Ballhausen, Prune Lagner, Julia Benusch, Anja Cichon, Annekathrin Zergiebel, Michel Oris, Katharina M. Schnitzspahn

**Affiliations:** ^1^Department of Psychology, University of GenevaGeneva, Switzerland; ^2^Center for the Interdisciplinary Study of Gerontology and Vulnerability and NCCR LIVES, University of GenevaGeneva, Switzerland; ^3^Department of Psychology, Technische Universität DresdenDresden, Germany

**Keywords:** prospective memory, age differences, laboratory testing situation, stress, relaxation

## Abstract

To explain age deficits found in laboratory-based prospective memory (PM) tasks, it has recently been suggested that the testing situation *per se* may be more stressful for older adults, thereby impairing their performance. To test this assumption, subjective and physiological stress levels were assessed at several times during the experiment in 33 younger and 29 older adults. In addition, half of participants were randomized in a condition where they completed a relaxation intervention before performing a time-based PM task. Results confirmed the age deficit in laboratory PM. Subjective and physiological stress levels showed no age difference and no detrimental association with PM. The intervention successfully reduced stress levels in both age groups but had no effect on PM or the age deficit. In conclusion, data suggest that age deficits usually observed in laboratory PM may not be due to higher stress levels in the older adults.

## Introduction

Prospective memory (PM) is defined as remembering to realize a planned intention at a particular moment in the future while being engaged in an ongoing activity (Brandimonte et al., 1996; Ellis, 1996; Ellis and Kvavilashvili, 2000). Everyday examples of PM are remembering to turn off the stove after cooking, paying the utility bills on time, or remembering to take medication according to a schedule. There is a conceptual distinction between time- and event-based PM tasks (Einstein and McDaniel, 1996). Time-based PM tasks require an individual to perform a specified behavior at a specific time or after the passage of a given amount of time, whereas event-based PM tasks require an individual to perform a specified behavior in response to an external cue (Einstein and McDaniel, 1990). For the past three decades, developmental psychologists have investigated whether performance on PM tasks declines in older age. In their meta-analytic review, Henry et al. (2004) concluded that older adults generally perform worse than younger adults in laboratory PM tasks, which holds for both time-based and event-based tasks (see Kliegel et al., 2008; Ihle et al., 2013, for more recent meta-analyses confirming this pattern).

Interestingly, Henry et al. (2004) also revealed that this age deficit turns into an age benefit when participants are tested in their everyday life, which has been termed “age PM paradox” (Rendell and Thomson, 1999; Rendell and Craik, 2000; Schnitzspahn et al., 2011). Following up on this pattern, research has been attempting to discover the key variables affecting this paradox. In this line, the present paper will test one recent hypothesis that has been put forward to possibly (at least partly) explain the robust PM age deficit in the laboratory: laboratory sessions being especially stressful for older adults and thereby hampering their performance. Reviewing the evidence on age differences in PM, Phillips et al. (2008) outlined the possibility that the higher performance of younger adults in the laboratory may be explained by younger adults’ (who are often students participating for course credit) greater experience with laboratory testing situations and cognitive tests. Similarly, they argue that laboratory PM tasks may be more stressful for older adults due to greater novelty of those testing procedures.

This prediction has only recently been tested to explain age-related cognitive deficits in the laboratory in general. Specifically, it has been suggested that older adults may be more stressed through contextual features of typical laboratory settings due to greater novelty and unpredictability (Sindi et al., 2013). To formally examine this assumption, Sindi et al. (2013) implemented two laboratory testing conditions (i.e., favoring younger vs. favoring older adults) and examined stress levels as well as immediate and delayed memory performance in younger and older adults. The two testing conditions differed with regard to several features (i.e., location, time of testing, age of experimenter, task type, and instruction): in the favoring young condition, the location was unfamiliar for older adults (i.e., at the university), the testing took place in the afternoon, the experimenter was a young student, the memory task was a word list recall, and the instructions made a strong emphasize on the memory component of the task. In contrast, in the favoring old condition, the location was familiar for older adults (i.e., at a health institute), the testing took place in the morning, the experimenter was an older research assistant, the memory task was a face association recall, and the instructions made only a weak emphasize on the memory component. Stress was assessed by measuring salivary cortisol levels at home before the testings and during the testings in the laboratory. Compared with baseline cortisol level measured at home, cortisol concentrations for younger and older adults were on a comparable level in the condition favoring the elderly. However, stress levels of older adults were increased in the testing condition favoring the young which actually represented a usual testing situation in cognitive aging research. Thus, this suggests that a traditional laboratory testing situation may indeed be more stressful for older adults. This is in line with the more general notion that tasks tend to evoke pronounced cortisol responses if they are uncontrollable or characterized by social-evaluative threat (where task performance could be negatively judged by others; Kirschbaum et al., 1993; Dickerson and Kemeny, 2004; Dijkstra et al., 2009), which may be likely the case for laboratory performance assessments. Concerning possible stress effects on memory performance, Sindi et al. (2013) showed that in the testing condition favoring the young, older adults’ forgetting rate in the delayed memory test was steeper compared to younger adults, suggesting that stress caused by the testing environment may enforce negative age effects. This suggestion dovetails with studies showing that the stress hormone cortisol negatively affects memory performance (e.g., Lupien et al., 1997; Lee et al., 2007) and more generally, that stress impairs cognitive functioning (e.g., Oei et al., 2006; Luethi et al., 2008; Liston et al., 2009; Qin et al., 2009) and links those effects to the laboratory test setting typically used in cognitive aging research, and also in most PM studies.

In consequence, in line with the suggestions of Sindi et al. (2013) one could raise the question whether the age deficit in laboratory PM performance is (partly) attributable to increased stress levels in older adults evoked by the nature of the laboratory testing situation *per se*. First evidence that stress can influence PM performance in the laboratory came from a study by Nater et al. (2006) on younger adults only showing that stress affected (though here enhanced) performance in a time-based PM task. Moreover, studies focusing on naturalistic PM suggest that perceived stress is negatively associated with PM performance (e.g., Schnitzspahn et al., 2011; Ihle et al., 2012). So far, no study has directly tested the inherent role of laboratory testing situations for evoking stress and its possible effects on age differences in laboratory PM.

Therefore, the present study aimed to investigate the role of stress levels caused by the testing situation in laboratory PM and for this purpose followed five major goals: (1) to examine whether the age deficit in laboratory PM can be confirmed with present data; (2) to evaluate whether the typical laboratory assessment of PM evokes higher stress levels in older compared to younger adults during the experiment; (3) to test whether stress levels are negatively associated with PM performance. In addition, as a novel exploration in this context, we intended to evaluate the effect of a relaxation intervention that is applied to reduce perceived and physiological stress levels. Specifically, it is examined; (4) whether this intervention can successfully reduce stress levels in the laboratory; and (5) whether it has an effect on PM performance in general and/or PM age differences.

## Method

### Participants

The sample consisted of 62 participants: 33 younger adults (mean age = 20.8 years, *SD* = 2.1, range: 18–26) and 29 older adults (mean age = 65.2 years, *SD* = 4.9, range: 54–74). All younger adults were undergraduate students from the Technische Universität Dresden, who volunteered in exchange for partial course credit. All older adults were volunteers. Among all participants, gift certificates for books were raffled. None of the participants had previous or current physical and mental health problems, intake of medication affecting cognitive functioning, or a recent critical incident (e.g., illness/disease of or dispute with a relative or close friend, etc. that may affect stress levels in general). The two age groups differed with respect to gender distribution (younger adults: 9% males; older adults: 41% males; *χ*^2^ (*df* = 1) = 7.10, *p* = 0.008).^1^ In terms of general cognitive abilities, the two age groups differed in both crystallized intelligence and processing speed in the anticipated direction. Crystallized intelligence was assessed with a German vocabulary test (MWT; Lehrl, 1977) in which older adults (*M* = 32.79, *SD* = 2.06) attained significantly higher scores than younger adults (*M* = 30.33, *SD* = 2.57; *t*_(60)_ = 4.12, *p* < 0.001). Processing speed was indexed using the Digit-Symbol-task (Wechsler, 1981), with younger adults obtaining significantly higher scores (*M* = 70.82, *SD* = 10.88) than older adults (*M* = 49.61, *SD* = 8.58; *t*_(59)_ = 8.34, *p* < 0.001). Both younger and older participants were randomly assigned to one of two experimental conditions: half of them were asked to perform a relaxation exercise supposed to reduce perceived stress levels as well as physiological arousal. The other half was assigned to a control condition (see Section Procedure for further details). All participants gave informed consent and the present study included adherence to the declaration of Helsinki.

### Materials

#### PM task

In the ongoing task, participants were presented with a 2-back working memory task. Names of cities in Germany were serially displayed in the center of the screen (each for 3000 ms, with an inter-stimulus interval of 1000 ms) in the same pseudo-randomized order for all participants. It was asked to decide whether or not the present city name has occurred two trials earlier by pressing either a green key for “Yes” or a red key for “No”. The task started with a practice phase. Then, a first test block (to assess baseline ongoing task performance) followed, which lasted 5 min and for which 25 out of the 75 stimuli were 2-back hit items. For the time-based PM task embedded in the second and third test block, participants were instructed to remember to press the “V” key every minute (counted from the beginning of the respective block—see e.g., Park et al., 1997; Kliegel et al., 2005; Kliegel and Jäger, 2006; Altgassen et al., 2010; Schnitzspahn et al., 2011, for similar procedures). Participants were told that they should remember to press the key on the keyboard “as exactly as possible” in the moment when the respective 1-min segment had elapsed. Participants could press the space bar to briefly monitor the elapsed time. A time window of 10 s (5 s before/after the target time) was used to classify responses as correct PM answers. Such a time window has been successfully used in previous PM research avoiding ceiling effects in younger and older adults (e.g., Park et al., 1997; Schnitzspahn et al., 2011).^2^ The instructions for the PM task were given after the ongoing task baseline block. Each participant was asked to explain the PM task in his/her own words, and if necessary, the instructions were discussed until the experimenter was confident that the participant understood the procedure. To ensure a delay between the PM task instructions and its execution, participants performed the relaxation exercise (or read an article in a control condition). Both PM blocks lasted 5 min and 5 s (to assure that the 5-min PM response did not coincide with the end of the block). Furthermore, each block contained five PM target times as well as 76 stimuli of which 25 were 2-back hit items. The ongoing and PM task performance scores were the number of correct responses across the two PM blocks (i.e., “Yes”/“No” for the 2-back working memory task and the “V” key for the PM task with maximum possible PM hits = 10).^3^

#### Stress measurements

Stress levels were measured at three times during the experiment: first, for a baseline assessment at the beginning of the testing session (i.e., before the ongoing task baseline block; pre), the second time after the relaxation intervention (or after reading the text, respectively; post), that is, directly before the PM task, and the third time directly after the PM task (follow-up). Subjective stress levels were assessed using a subscale of a multidimensional state questionnaire (MDBF; Steyer et al., 1997). Participants rated their current state in terms of feeling restless, feeling calm, feeling troubled, feeling relaxed, feeling balanced, feeling tense, feeling nervous, and feeling tranquil, with the help of eight items, each based on a 5-point Likert scale ranged from 1 (“not at all”) to 5 (“very”). Item scores of items representing a “non-stressed” state were reversed before all scores were summed up. In addition, as a measure of subjective stress level that refers to the PM task itself, we asked participants after the PM task to evaluate retrospectively whether they found this task stressful, based on a 5-point Likert scale ranged from 1 (“not at all”) to 5 (“very”). Moreover, physiological arousal in terms of heart rate as a further indicator for stress level (e.g., Kudielka et al., 2004) was measured using a heart rate monitor. It consisted of a strap that was put around the chest and a monitor watch. Each time after the participants had filled out the MDBF questionnaire, the experimenter noted the current heart rate (thus also at three times during the experiment).

### Procedure

Both younger and older adults were individually tested in the laboratory of developmental psychology at the Technische Universität Dresden. After participants gave their consent for the experiment, they were asked whether they were wearing a wrist watch and if so, to remove it for the session (to not have any hint during the time-based PM task). Next, baseline stress levels were measured. Then, participants filled out a socio-demographic questionnaire and the ongoing task baseline block followed. Next, participants received the instructions for the PM task. After that, half of the participants performed a relaxation exercise while the other half read a text. The instructions for the relaxation exercise were read aloud by the experimenter. The relaxation comprised elements of progressive muscle relaxation (PMR) and autogenic training (AT). Both methods are well-established for reducing subjective and physiological indices of stress (e.g., Shoemaker and Tasto, 1975; Kanji and Ernst, 2000; Rohrmann et al., 2001; Pawlow and Jones, 2002, 2005). In the meantime, the control group had to read an article on Albert Einstein and answer questions about its content. Note that the text did not contain information about the investigated constructs (i.e., stress, relaxation, or memory). Both activities, the relaxation exercise and reading the text, lasted about 10 min. This was followed by the second measurement of stress levels. Then, the two PM test blocks were executed, and directly afterwards the third measurement of stress levels followed (including also the retrospective stress rating regarding the PM task itself). Finally, the measures of processing speed and crystallized intelligence were assessed. To close the testing session, all participants were debriefed and the goals of the present study were explained in detail.

### Statistical analyses

Repeated measures of stress levels were analyzed using a 2 × 2 × 3 ANOVA with the between-subject factors age (young, old) and relaxation condition (relaxation, control) and the within-subject factor measurement time (pre, post, follow-up). Prospective memory performance, time monitoring, ongoing task performance, and the single stress rating regarding the PM task itself were analyzed using a 2 × 2 ANOVA with the between-subject factors age (young, old) and relaxation condition (relaxation, control).

Note that to account for the age-related decrease in heart rate across the lifespan (e.g., Kudielka et al., 2004) when examining physiological parameters such as heart rate in different age groups, it is usual not to compare current levels but instead current levels in relation to baseline level (i.e., in terms of a difference or an interaction). Thus, to investigate group differences regarding the physical response (in relation to baseline level), we tested for interaction effects of group by measurement time. To evaluate associations of PM/ongoing task performance/monitoring with physiological stress levels, we calculated the difference between current and baseline heart rate as an indicator for the physical response (in relation to baseline level).

In all ANOVAs with more than two repeated measures, Mauchly’s Test of Sphericity was used to test the assumption of sphericity. If the assumption was violated, the Huynh–Feldt corrected *F*-statistic was calculated (all *ɛ* > 0.84). For all analyses, the R environment was used (version 3.0.3; R Development Core Team, 2014).

## Results

### Stress levels

**Figure 1:**
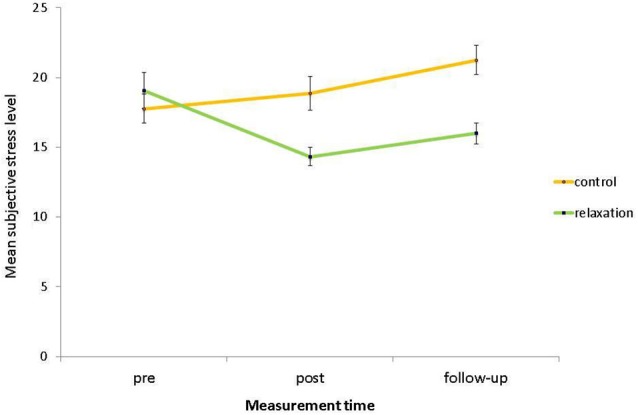
**Mean subjective stress levels, i.e., baseline assessment (pre), after the intervention (post), and after the PM task (follow-up) as a function of relaxation condition**. Bars represent standard errors.

For subjective stress levels, there was a significant main effect of relaxation condition, *F*_(1,58)_ = 5.84, *p* = 0.019, *η*^2^ = 0.09, with lower stress levels in the relaxation group (*M* = 16.47, *SD* = 4.29) than in the control group (*M* = 19.29, *SD* = 5.14). Furthermore, there was a significant main effect of measurement time, *F*_(2,116)_ = 4.78, *p* = 0.015, *η*^2^ = 0.08, with lower stress levels in the post phase (*M* = 16.68, *SD* = 5.97) than in the pre phase (*M* = 18.39, *SD* = 6.46) and on follow-up (*M* = 18.71, *SD* = 5.69). Most importantly to test for the relaxation intervention effect, there was a significant interaction of relaxation condition with measurement time, *F*_(2,116)_ = 13.00, *p* < 0.001, *η*^2^ = 0.18. Subsequent contrasts revealed that this interaction was evident between the pre and the post phase, *F*_(1,58)_ = 15.65, *p* < 0.001, *η*^2^ = 0.21, indicating that the stress reduction was successful (see Figure 1). This significant interaction held also when comparing the pre phase with follow-up, *F*_(1,58)_ = 16.06, *p* < 0.001, *η*^2^ = 0.22, and was not significant between post phase and follow-up, *F*_(1,58)_ = 0.52, *p* = 0.474, *η*^2^ < 0.01, indicating that the stress reduction in the post phase held until follow-up. Moreover for the overall ANOVA, there was no significant main effect of age, *F*_(1,58)_ = 0.86, *p* = 0.358, *η*^2^ = 0.01, indicating that stress levels of younger (*M* = 18.42, *SD* = 4.53) and older adults (*M* = 17.36, *SD* = 5.35) did not differ. There was a significant interaction of age group with measurement time, *F*_(2,116)_ = 3.72, *p* = 0.035, *η*^2^ = 0.06. This result is driven by the fact that younger adults started with a relatively high baseline subjective stress level (*M* = 19.88, *SD* = 6.82) but then reached at post-intervention assessment (*M* = 16.52, *SD* = 5.33) almost identical values as older adults in both phases (pre: *M* = 16.69, *SD* = 5.68; post: *M* = 16.86, *SD* = 6.72). Thereby, there was a trend for higher baseline subjective stress level in younger compared to older adults, *F*_(1,60)_ = 3.94, *p* = 0.052, *η*^2^ = 0.06. There were no age differences in stress level in the post phase, *F*_(1,60)_ = 0.05, *p* = 0.822, *η*^2^ < 0.01. Also on follow-up, both age groups showed almost identical values (younger: *M* = 18.88, *SD* = 5.18; older: *M* = 18.52, *SD* = 6.30; *F*_(1,60)_ = 0.06, *p* = 0.805, *η*^2^ < 0.01). Importantly, the interactions of age group by relaxation condition and of age group by relaxation condition by measurement time were non-significant (*p*s > 0.248), indicating that the intervention did not favor any age group.

For the additional retrospective stress rating regarding the PM task itself, there was no main effect of age, with younger adults (*M* = 2.82, *SD* = 1.10) and older adults (*M* = 2.90, *SD* = 1.21) showing comparable subjective stress levels, *F*_(1,58)_ = 0.06, *p* = 0.802, *η*^2^ < 0.01. There was a trend of lower perceived stress levels in the relaxation group (*M* = 2.60, *SD* = 1.19) than in the control group (*M* = 3.09, *SD* = 1.06; *F*_(1,58)_ = 2.98, *p* = 0.090, *η*^2^ = 0.05). However, there was no interaction of age group with relaxation condition, *F*_(1,58)_ = 0.19, *p* = 0.664, *η*^2^ < 0.01.

**Figure 2:**
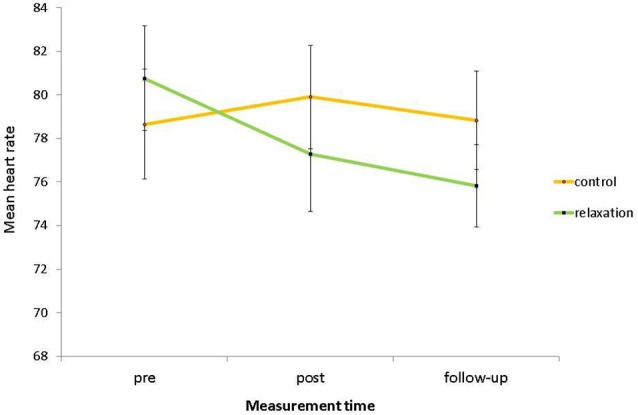
**Mean heart rate, i.e., baseline assessment (pre), after the intervention (post), and after the PM task (follow-up) as a function of relaxation condition**. Bars represent standard errors.

For heart rates, there was no significant main effect of relaxation condition, *F*_(1,58)_ = 0.12, *p* = 0.726, *η*^2^ < 0.01, indicating that, collapsed across the three measurement points, heart rates of the relaxation group (*M* = 77.97, *SD* = 11.58) and the control group (*M* = 79.14, *SD* = 12.59) did not differ. Furthermore, there was no significant main effect of measurement time, *F*_(2,116)_ = 2.20, *p* = 0.116, *η*^2^ = 0.04, indicating that, collapsed across both conditions, heart rates in the pre phase (*M* = 79.68, *SD* = 13.64), in the post phase (*M* = 78.65, *SD* = 13.89), and on follow-up (*M* = 77.39, *SD* = 11.69) did not differ. However, most importantly to test for the relaxation intervention effect, there was a significant interaction of relaxation condition with measurement time, *F*_(2,116)_ = 3.10, *p* = 0.049, *η*^2^ = 0.05. Subsequent contrasts revealed that this interaction tend to emerge between the pre and the post phase, *F*_(1,58)_ = 3.69, *p* = 0.060, *η*^2^ = 0.06, indicating again that the stress reduction was successful (see Figure 2). The interaction was significant when comparing the pre phase with follow-up, *F*_(1,58)_ = 5.01, *p* = 0.029, *η*^2^ = 0.08, and was not significant between post phase and follow-up, *F*_(1,58)_ = 0.06, *p* = 0.810, *η*^2^ < 0.01, indicating that the stress reduction in the post phase held until follow-up. Furthermore for the overall ANOVA, there was a main effect of age, *F*_(1,58)_ = 16.55, *p* < 0.001, *η*^2^ = 0.22, with older adults (*M* = 72.60, *SD* = 9.81) showing a significantly lower heart rate than younger adults (*M* = 83.82, *SD* = 11.45). Note that this finding reflects the age-related decrease of heart rate across the lifespan (e.g., Kudielka et al., 2004). Importantly in this context of comparing physiological parameters in different age groups, there was no significant interaction of age group with measurement time, *F*_(2,116)_ = 0.05, *p* = 0.949, *η*^2^ < 0.01, indicating that both age groups showed a comparable physical response to the testing situation (in relation to baseline level). Furthermore, there were no significant interactions of age group by relaxation condition and of age group by relaxation condition by measurement time (*p*s > 0.194), again indicating that the intervention did not favor any age group.

### PM, time monitoring, and ongoing task performance

**Figure 3:**
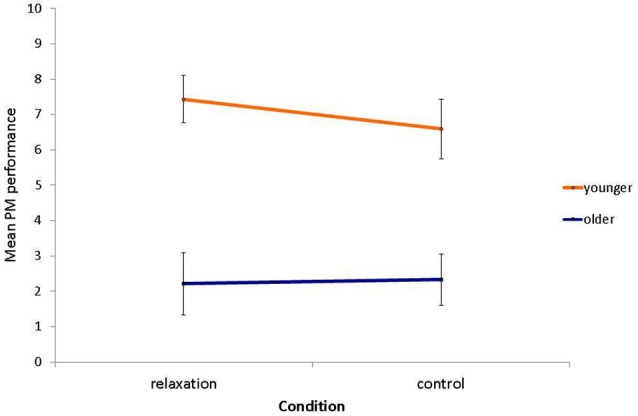
**Mean PM performance as a function of relaxation condition and age (max. possible PM hits = 10)**. Bars represent standard errors.

The age deficit in laboratory PM performance was confirmed, *F*_(1,58)_ = 36.14, *p* < 0.001, *η*^2^ = 0.38, with younger adults (*M* = 7.00, *SD* = 3.10) attaining significantly more PM hits than older adults (*M* = 2.28, *SD* = 3.01; see Figure 3). However, there was neither an effect of relaxation condition (relaxation group: *M* = 5.00, *SD* = 3.96; control group: *M* = 4.59, *SD* = 3.81; *F*_(1,58)_ = 0.21, *p* = 0.645, *η*^2^ < 0.01), nor an interaction with age group, *F*_(1,58)_ = 0.38, *p* = 0.542, *η*^2^ < 0.01.

For time monitoring (i.e., number of presses of the space bar, summed up across both PM blocks), there was a main effect of age, *F*_(1,58)_ = 21.64, *p* < 0.001, *η*^2^ = 0.27, with younger adults (*M* = 19.85, *SD* = 14.57) monitoring the clock significantly more often than older adults (*M* = 5.72, *SD* = 7.89). However, there was neither an effect of relaxation condition (relaxation group: *M* = 13.27, *SD* = 14.19; control group: *M* = 13.22, *SD* = 13.65; *F*_(1,58)_ < 0.01, *p* = 0.950, *η*^2^ < 0.01), nor an interaction with age group, *F*_(1,58)_ = 1.15, *p* = 0.288, *η*^2^ = 0.02.

For ongoing task performance (i.e., number of correct responses, averaged for both blocks), there was a main effect of age, *F*_(1,58)_ = 9.62, *p* = 0.003, *η*^2^ = 0.14, with younger adults (*M* = 67.56, *SD* = 7.13) attaining significantly more correct responses than older adults (*M* = 62.29, *SD* = 6.15). However, there was neither an effect of relaxation condition (relaxation group: *M* = 63.78, *SD* = 8.56; control group: *M* = 66.33, *SD* = 5.35; *F*_(1,58)_ = 2.25, *p* = 0.139, *η*^2^ = 0.04), nor an interaction with age group, *F*_(1,58)_ < 0.01, *p* = 0.976, *η*^2^ < 0.01.

### Correlations of stress levels with PM, time monitoring, and ongoing task performance

Higher baseline subjective stress level was significantly related to better PM performance and better time monitoring (overall and in older, but not in younger adults). In addition, higher heart rate (on follow-up and on a one tailed-level in the post phase) was significantly associated with better time monitoring in older adults only. There were no other significant correlations of subjective stress level, heart rate, or the retrospective stress rating regarding the PM task itself with PM performance, time monitoring, or ongoing task performance (see Table 1 for the full correlation matrix). More frequent time monitoring was significantly correlated with better PM performance (overall and in both age groups separately).

**Table 1:** **Correlations between measures**.

Variable	*1*	*2*	*3*	*4*	*5*	*6*	*7*	*8*
				**Overall**
1. Subjective stress (pre)	—
2. Subjective stress (post)	0.44^***^	—
3. Subjective stress (follow-up)	0.31^*^	0.76^***^	—
4. Subjective stress (PM task)	0.09 ns	0.36^**^	0.43^***^	—
5. Heart rate (post-pre)	0.02 ns	0.07 ns	0.14 ns	−0.13 ns	—
6. Heart rate (follow-up-pre)	0.04 ns	0.15 ns	0.18 ns	0.17 ns	0.63^***^	—
7. Ongoing task performance	0.06 ns	0.02 ns	0.003 ns	−0.14 ns	0.14 ns	−0.02 ns	—
8. PM performance	0.29^*^	0.07 ns	0.11 ns	0.17 ns	0.06 ns	0.11 ns	0.14 ns	—
9. Monitoring	0.30^*^	−0.01 ns	−0.01 ns	−0.001 ns	0.15 ns	0.10 ns	0.20 ns	0.71^***^
				**Younger adults**
1. Subjective stress (pre)	—
2. Subjective stress (post)	0.42^*^	—
3. Subjective stress (follow-up)	0.22 ns	0.67^***^	—
4. Subjective stress (PM task)	0.03 ns	0.27 ns	0.43^*^	—
5. Heart rate (post-pre)	0.05 ns	0.12 ns	0.24 ns	−0.13 ns	—
6. Heart rate (follow-up-pre)	0.05 ns	0.05 ns	0.18 ns	0.22 ns	0.60^***^	—
7. Ongoing task performance	0.05 ns	0.15 ns	−0.08 ns	−0.24 ns	0.20 ns	−0.01 ns	—
8. PM performance	−0.03 ns	−0.05 ns	0.11 ns	0.27 ns	0.04 ns	0.06 ns	−0.05 ns	—
9. Monitoring	0.15 ns	−0.10 ns	−0.06 ns	0.03 ns	0.12 ns	−0.003 ns	0.04 ns	0.56^***^
				**Older adults**
1. Subjective stress (pre)	—
2. Subjective stress (post)	0.53^**^	—
3. Subjective stress (follow-up)	0.42^*^	0.83^***^	—
4. Subjective stress (PM task)	0.19 ns	0.44^*^	0.43^*^	—
5. Heart rate (post-pre)	−0.10 ns	−0.002 ns	−0.04 ns	−0.16 ns	—
6. Heart rate (follow-up-pre)	0.02 ns	0.34’	0.19 ns	0.12 ns	0.76^***^	—
7. Ongoing task performance	−0.16 ns	−0.09 ns	0.07 ns	−0.01 ns	−0.06 ns	−0.09 ns	—
8. PM performance	0.48^**^	0.26 ns	0.13 ns	0.20 ns	0.12 ns	0.23 ns	−0.22 ns	—
9. Monitoring	0.39^*^	0.19 ns	0.03 ns	0.01 ns	0.35’	0.43^*^	−0.04 ns	0.71^***^

*Note. Pre = baseline assessment; post = after the intervention; follow-up = after the PM task. Subjective stress level (PM task) = retrospective stress rating regarding the PM task itself*.

*** p < 0.001; ** p < 0.01; * p < 0.05; ’ p < 0.10, significance at one-tailed level; ns = non-significant (i.e., p > 0.10).

## Discussion

The present study investigated the role of stress on age-related laboratory PM performance and addressed five major questions: first, evaluating age differences, results confirmed the age deficit in laboratory PM with present data. Second, it was examined whether the laboratory testing situation *per se* evoked stress particularly in older participants, which would result in higher stress levels in older compared to younger adults during the experiment. Yet, results indicated that older adults did not show higher stress levels compared to younger adults at any time measured during the experiment. Subjective stress level at baseline was even higher in younger compared to older adults. In addition, there were no age differences in the retrospective stress ratings concerning the PM task itself. Third, it was tested whether stress levels were negatively correlated with performance in the PM task. Results showed that subjective stress levels and heart rate (while showing the predicted pattern across the relaxation and testing situation) did not show a significant negative association with PM performance, time monitoring, or ongoing task performance. Yet there were some few significant, but positive relations of the physical response to the testing situation in terms of heart rate with time monitoring in older (but not younger) adults as well as of baseline subjective stress level with PM performance and time monitoring (but again not for younger adults). Importantly, the stress rating concerning the PM task itself was not related to PM performance, time monitoring, or ongoing task performance. Fourth, a relaxation intervention was applied (to half of the participants) to reduce perceived and physiological stress levels. Following this experimental manipulation, it was tested whether the stress level reduction was successful. Results showed that after relaxation, subjective stress levels and heart rate were significantly reduced. Finally, it was evaluated whether the intervention had an effect on performance in the PM task. Yet, results showed that the intervention had neither an effect on PM, ongoing task performance, or time monitoring in general nor on the age deficit in any of these cognitive measures in particular. All in all, present data provide no evidence that laboratory PM performance is detrimentally influenced by individuals’ stress level and suggest that—if the nature of the laboratory setting *per se* should evoke the PM age deficit—this is possibly not due to increased levels of stress in older adults.

In light of the evidence that stress seems to impair a variety of cognitive functions such as attentional control and working memory (e.g., Oei et al., 2006; Luethi et al., 2008; Liston et al., 2009; Qin et al., 2009), the present finding of no negative association between stress levels and PM does not support this general view. However, the literature on stress effects on PM performance is mixed: while two studies on naturalistic PM in younger and older adults report a negative association of perceived stress and PM (Schnitzspahn et al., 2011; Ihle et al., 2012), Nater et al. (2006) found a positive effect of stress on time-based laboratory PM in younger adults. However, Nater et al. (2006) found no effect on event-based PM. Also studying younger adults only, Nakayama et al. (2005) found as well no association of stress levels with event-based PM performance. Recently, Walser et al. (2013) experimentally assigned younger adults to a stress vs. a no-stress control group. For stress induction, they used the Trier Social Stress Test (TSST; Kirschbaum et al., 1993), which evokes acute psychosocial stress in a standardized procedure. Following this manipulation, both experimental groups performed an event-based PM task. Although the stress group showed elevated levels of salivary cortisol throughout the cognitive testing period compared to the no-stress group, PM performance did not differ between groups. They concluded that cognitive control processes underlying PM intention retrieval may be mostly preserved even under conditions of acute stress. Due to the very small number of studies available, it remains an open question whether effects of stress may differ between naturalistic and laboratory PM as well as time-based and event-based PM. Yet, considering these latter three results from laboratory studies, in line with the present findings, it could be suggested that there may be no universal and generally strong detrimental influence of individuals’ stress level on (age-related) laboratory PM. Moreover, all in all, the present findings do not support the suggestions of Sindi et al. (2013) that memory age deficits usually found in laboratory settings could be caused by increased stress in older adults; at least for a traditional time-based PM task.

At this point it remains an open question whether laboratory PM is an exception in light of the suggestions of Sindi et al. (2013). However, Sindi et al. (2013) manipulated several contextual features such as the location of testing, testing time, age of the experimenter, task instructions, and characteristics of the task itself to construct the two different testing conditions (favoring young vs. favoring old). Regarding the potential influence of the testing location, we did not find higher stress levels in older compared to younger adults at any time measured during the experiment. This is remarkable given that both younger and older adults had been tested in the laboratory at the university. This is in sharp contrast to the findings of Sindi et al. (2013) who found pronounced stress responses in older adults when they were tested at the university. This underlines that being tested in a laboratory at the university (as common in studies on cognitive aging) *per se* may not be a critical aspect evoking stress in older adults and thereby causing age deficits in cognitive functioning. Hence, the finding of Sindi et al. (2013). that age differences in cognitive performance varied as a function of the testing situation may just as well be traced back to differences in other contextual features in the applied testing environment (see, for example, the strong evidence of time of day effects on age-related cognitive performance; May and Hasher, 1998; Borella et al., 2011). In other words, besides differences in stress levels, there might be more factors contributing to the age deficit in cognitive functioning and particularly in laboratory PM performance. Factors in the testing environment that are currently considered to influence age differences in laboratory PM concern for example the social interaction with the experimenter (Altgassen et al., 2010), motivational aspects linked to the personal importance of the PM task or the participation itself (Phillips et al., 2008), or characteristics of the task implementation determining how much older adults can benefit from strategies and knowledge acquired in everyday life (Schnitzspahn et al., 2011). A further research line that may help to explain PM age differences predicts that in general, older adults may (have to) exhibit greater levels of effort to perform on cognitive tasks, which may be reflected in subjective perceptions of difficulty. In this context, it has been shown that costs of performing on cognitive tasks increase with age in adulthood, and that these costs influence individuals’ willingness to engage resources in support of demanding cognitive activities (e.g., Hess and Ennis, 2012; Ennis et al., 2013). Although potentially relevant when discussing cognitive age differences, to our knowledge no study so far has explicitly targeted this issue as an additional mechanism that could (at least partly) explain the PM age deficit usually observed in the laboratory.

Limitations of the present study concern the following issues: First, it is possible that the effect of the relaxation intervention on stress levels was diminished as both younger and older participants showed rather moderate levels of stress when they began the experiment. Thus, the variance to further reduce it was restricted (which might also be partly responsible for the low correlations between individual differences in stress levels and PM performance). Additionally, the question could be raised whether a reduction of stress levels through relaxation concerns a different type of stress than acute stress induced in the laboratory. However, due to the assumption of increased stress levels in older adults evoked by the laboratory setting *per se*, we intended not to (further) increase stress levels even more but to reduce it to achieve a relatively low stress level in both age groups for the testing. Results showed that the reduction and hence the experimental manipulation was successful. Yet, we did not find that the stress reduction affected PM performance. Note that this is in line with the findings of Walser et al. (2013) who induced stress but did not find an effect on PM as well.

Second, as there are no normative data for stress levels in the laboratory, it remains an open question whether the findings of the present study may be influenced by the possibility that stress levels of older adults were lower (or respectively that stress levels of younger adults were higher) than usual. However, findings showed that there were no age differences regarding the relaxation effect meaning that both groups benefited equally from the relaxation and hence showed comparable stress reductions which would not be the case if the aforementioned pattern would be true (i.e., a smaller reduction in older adults due to lower initial stress levels and similarly a larger reduction in younger adults due to higher initial stress levels). Additionally, one could argue that physical stress levels of younger and older adults were not equal as older adults showed a significantly lower heart rate. Note that this finding reflects the age-related decrease in heart rate across the lifespan (e.g., Kudielka et al., 2004). Hence, to study age differences in physiological parameters such as heart rate, it is usual not to compare current levels but instead current levels in relation to baseline level, i.e., in terms of a difference or an interaction, as applied in the reported analyses: there was no significant interaction of age group with measurement time, indicating that both age groups showed a comparable physical response (in relation to baseline level). Furthermore, there were no significant interactions of age group by relaxation condition and of age group by relaxation condition by measurement time, indicating that the intervention did not favor any age group. To evaluate associations with physiological stress levels, we calculated the difference between current and baseline heart rate (i.e., post/follow-up minus pre) as an indicator for the physical response (in relation to baseline level), revealing no significant negative correlation with PM/ongoing task performance or time monitoring (see above for further details).

Third, the concern could be raised that heart rate is not sensitive enough as a reliable indicator for physiological stress in this context. Indeed, the relaxation intervention was less effective in reducing the heart rate compared with the effects on the subjective stress levels. Nevertheless, a significant relaxation effect could be detected for both stress measures. Yet, present findings await replication with other or multiple indicators for physiological stress such as blood pressure, skin conductance, or salivary cortisol level. It may be also of interest to compare whether there are differences between indicators that reflect the activity of the sympathetic system (such as heart rate, as in the present study) and those that reflect the activity of the endocrine system (such as cortisol, as in Sindi et al., 2013). Moreover, we evaluated the possibility that not the test setting *per se* (as proposed by Sindi et al., 2013) but the PM task itself is stressful, and maybe to a different extent in older than younger adults (see e.g., Kirschbaum et al., 1993; Dickerson and Kemeny, 2004; Dijkstra et al., 2009, for effects of stressful tasks in general). Although we can not exclude the possibility that there were fluctuations of stress levels with higher peaks during the PM task than directly before and afterwards (where stress levels had been assessed), results indicated that stress levels did not change substantially between post treatment assessment and follow-up directly after the PM task (for both age groups) and suggest that stress levels remained stable during the PM task. In addition, there were no age differences in retrospective stress ratings concerning the PM task itself. However, in future research, physiological stress indicators should be assessed continuously during the PM task so that fluctuations and stress peaks could be investigated during PM performance, and in particular, whether this differs in younger and older adults.

Fourth, we acknowledge that the older participants were relatively young in this study. Thus, future research may investigate whether present findings of no detrimental associations of stress levels with (age-related) PM can be replicated with old-old adults. Likewise, it would be interesting to see whether present results hold for a broader variety of (time- and event-based) PM tasks assessed in the laboratory and a more naturalistic setting. Finally, one may argue that our null findings are due to low statistical power. Two things should be noted. First, we chose our sample size according to Sindi et al. (2013), which would have been sufficient to replicate their reported associations of stress evoking factors with cognitive performance. Second, a power analysis showed that with an alpha probability of 0.05 and a power of 0.80, we would have been able to detect effects of medium to large size when comparing the two experimental groups, and of medium size for correlations based on the overall sample. Thus, we acknowledge that the present power may have been too low to detect the very small associations of stress with PM performance in the present study. However, large stress effects (for which the present power was sufficient) seem unlikely. In particular, we underline that present associations of stress with PM performance are only of marginal size. Especially compared to the strong PM age effect observed (which is even larger than usually found with time-based laboratory PM tasks, i.e., *r* = −0.39, as reported in Henry et al., 2004, which corresponds to an *η*^2^ of 0.15), present stress effects (irrespective of their significance/non-significance) remain negligible.

## Conclusion

The present study suggests that age deficits usually observed in laboratory PM may not be due to higher stress levels in the older adults, thereby clarifying an important issue in the ongoing conceptual debate of underlying mechanisms in adult age differences in prospective remembering. Beyond that, as present results do not support the view that age deficits in the laboratory in general might be evoked by increased stress in older adults by the testing situation *per se*, they make an important contribution to cognitive aging research with respect to the fundamental question whether cognitive deficits in the laboratory reflect “true” performance failures.

## Conflict of interest statement

The authors declare that the research was conducted in the absence of any commercial or financial relationships that could be construed as a potential conflict of interest.

## Footnotes

^1^There was neither a main effect of gender nor an interaction with age on PM performance, time monitoring, or ongoing task performance (all *p*s > 0.302).

^2^For additional analyses, we also applied time windows of +/−2.5 s and +/−10 s to score PM responses as correct (see e.g., Park et al., 1997; for a similar approach). These analyses revealed the same pattern of results.

^3^For all individuals, performance in the ongoing task was greater than 50% assuring that they had to share resources between the ongoing and the PM task. Furthermore, after the experiment, all participants were able to recall what they had to do in the PM task.
